# COVID-19 in Children: A Review and Parallels to Other Hyperinflammatory Syndromes

**DOI:** 10.3389/fped.2020.593455

**Published:** 2020-11-24

**Authors:** Charlotte V. Hobbs, Alka Khaitan, Brian M. Kirmse, William Borkowsky

**Affiliations:** ^1^Division of Infectious Disease, Department of Pediatrics, Batson Children's Hospital, University of Mississippi Medical Center, Jackson, MS, United States; ^2^Department of Microbiology, University of Mississippi Medical Center, Jackson, MS, United States; ^3^Department of Pediatrics, The Ryan White Center for Pediatric Infectious Disease and Global Health, Indiana University School of Medicine, Indianapolis, IN, United States; ^4^Division of Medical Genetics, Department of Pediatrics, Batson Children's Hospital, University of Mississippi Medical Center, Jackson, MS, United States; ^5^Division of Infectious Diseases, Department of Pediatrics, New York University Langone Health, New York, NY, United States

**Keywords:** COVID-19, SARS-CoV-2, children, immune response, genetics, multisystem inflammatory

## Abstract

During the COVID-19 pandemic, children have had markedly different clinical presentations and outcomes compared to adults. In the acute phase of infection, younger children are relatively spared the severe consequences reported in adults. Yet, they are uniquely susceptible to the newly described Multisystem Inflammatory Syndrome in Children (MIS-C). This may result from the developmental “immunodeficiency” resulting from a Th2 polarization that starts *in utero* and is maintained for most of the first decade of life. MIS-C may be due to IgA complexes in a Th2 environment or a Th1-like response to COVID-19 antigens that developed slowly. Alternatively, MIS-C may occur in vulnerable hosts with genetic susceptibilities in other immune and non-immune pathways. Herein, we present a brief overview of the host immune response, virologic and genetic factors, and comparable inflammatory syndromes that may explain the pathophysiology leading to drastic differences in clinical presentation and outcomes of COVID-19 between children and adults.

## Introduction

SARS-CoV-2, a novel coronavirus, was first reported in December 2019 from Wuhan, China. The disease caused by SARS-CoV-2, COVID-19, subsequently spread in pandemic fashion over the following 10 months causing a wide spectrum of clinical symptoms from asymptomatic disease to death (1). Mortality correlated with preexisting inflammatory conditions including obesity, hypertension, sex (mortality rates in males 1.5 higher than in females), and most notably, age (2).

The natural history of COVID-19 in children has been different. In adults, initial reports of case fatality rates approached 15% compared to <1% in children (3). An initial report of a Chinese cohort of 36 children with documented COVID-19 infection revealed relatively mild symptoms (4). A larger cohort of 110 children documented mostly asymptomatic infection with a shorter duration of viral detection (11 vs. 17 days). These early reports indicated that symptomatic children were more likely to have fever, pneumonia, and lymphopenia (5).

Considerable information on COVID-19 is already available based on review of clinical and laboratory data, allowing for molecular, epidemiological, physiologic and clinical disease characterization. Given the information we have thus far, we construct hypotheses to explain some of the variation in infection and clinical outcomes of COVID-19 in children. This report attempts to apply our knowledge of virology, developmental immunology, comparable inflammatory syndromes, and host genetics to explain the drastic differences in clinical presentation and outcomes between children and adults.

## Origins and Transmission

Molecular sequencing identified SARS-CoV-2 as a relative of the SARS Coronavirus, most closely related to a bat coronavirus (6). Spread to humans may have occurred via an intermediate host, possibly the pangolin, and perhaps in the setting of exotic food markets (7). Experience from Wuhan suggested viral doubling time was every 2–3 days early in the epidemic (8), and the infection soon was recognized in Europe (9). It is now recognized that viral evolution has resulted in strains dominant in different parts of the world with amino acid mutations that impact infectivity (10).

Using surveillance and contact tracing, earlier studies suggested that children appear to acquire COVID-19 infection at a rate similar to that seen in adults (11). However, other studies document a risk 30% of that seen in exposed adults (12), and more recent mathematical modeling studies based on available epidemiologic data suggest that under age 20, susceptibility to infection is half that of those over age 20 (13). In contrast, a study indicated higher viral loads (lower threshold concentrations on RT PCR nasopharyngeal specimens) in children <5 years of age with mild to moderate COVID-19 (14). Children have been studied as index cases in households, and some studies show that children over age 10 transmit COVID-19 at rates similar to adults (15), but other studies with slightly different age breakdowns suggest this is not the case (16). Indeed, with few exceptions, while children may not suffer from significant disease with COVID-19, their role in transmission remains to be clarified (17), although recent meta-analyses suggest children have a lower risk of being infected with SARS CoV2 and “play a lesser role than adults in transmission at a population level (18).” These factors have significant implications for back-to-school programs for children and need to be evaluated carefully in the context of existing data and return-to-school programs ongoing in other countries, such as Denmark. Indeed, a recent study in Germany suggested that child to child transmission, in the context of reduced class size, face mask wearing (inside), exclusion of sick children, and with frequent ventilation of rooms, even with limited physical distancing among children, resulted in the maintenance of low COVID-19 transmission rates in children (19), and summer camps with stricter COVID-related measures (20). Nonetheless, transmission from children infected in daycare settings has been demonstrated (21). In addition, with heterogeneous approaches and persistently high levels of community transmission in the context of schools returning to in-person learning, such as is the current situation in states like Mississippi (Hobbs, C. pers. comm.), other locations will continue to suffer higher rates of pediatric COVID-19 infection (22).

## Receptor Presence

The major host target for SARS-CoV-2 is the ACE type 2 receptor (ACEr2). This receptor target distinguishes SARS-CoV-2 from bat coronaviruses, SARS and MERS. Infection of pulmonary and other epithelial cell types is facilitated by a protease, particularly a membrane embedded cysteine cell protease, TMPRSS2 (23). The viral entry receptor, ACEr2 is expressed mainly in lung type 2 alveoli but also in some nasal epithelial cells, and there is limited expression on pulmonary alveolar cells (24). It is also present in blood vessels, kidneys, cardiac and neural tissue (25). It has been speculated that younger children suffer less severe acute COVID-19 due to reduced ACEr2 expression (26), although recent studies have shown younger children have lower ACEr2 expression but not viral load (27). Expression is a function of clinical states (e.g., hypertension) and the presence of TMPRSS2 (a serine protease encoded on Chromosome 21) which increases ACEr2 expression, allows the virus to enter the cell after cleavage. Recently, it has been shown that COVID-19 can also infect small intestinal enterocytes via TMPRSS2 and TMPRSS4, another serine protease which facilitates fusogenic function of the virus (28). Androgen levels increase the protease expression (29), perhaps explaining the increase in infection among males. Importantly, ACEr2 gene expression is lower in children (30).

## Early Innate Immune Responses

Innate responses are variable in early life and generally below what is seen in adults, but likely sufficient to deal with low levels of virus seen early in infection. Early antiviral defense is mediated by the innate immune system with a variety of extracellular RNA sensors (TLRs) and intracellular sensors (IFIH1/MDA5, ZBP1, DDX58, RIG-1) to elicit a protective response. These innate defenses are genetically regulated and vary across different species (31). A variety of cytokines and chemokines, often under genetic control, would be expected to activate inflammatory responses (STAT1 or STAT3) that could eliminate low-level infection. This involves innate immune natural killer cells and polymorphonuclear cells. A recent study by transcriptomics highlights the importance of activated dendritic and neutrophils in the respiratory tract of subjects with COVID-19 (32). Viral antigens have been shown to subvert these responses. Innate immune responses (particularly IFNλ or IL28/29), play a significant role in determining the likelihood of developing bronchiolitis after RSV infection (33). One study showed that an interferon response element (IFTM3) is associated with excessive immune response to virus in an age dependent manner (34). Interferon lambda, a cytokine in the IL-10 family of cytokines (35) made by epithelial and dendritic cells, plays a significant role in defense against low levels of respiratory viruses. It binds to IFNLR1 and IL10RB, eliciting responses different from that of IFN1/2 (36). Different allelic variants of an IFN lambda SNP affect the outcome of Dengue infection in children, suggesting such genetic variation may be at play with other RNA viruses (37).

## Later Adaptive Immune Responses

If early immune responses fail to eliminate virus, adaptive responses are initiated to interfere with pulmonary infection. Local mucosal responses precede pulmonary responses and might be capable of eliminating virus sufficiently to modify pulmonary disease. A recent publication suggests that T cell trafficking to different target organs is highly associated with sex and age, perhaps determining where inflammation will occur (38). Although lymphocytopenia is associated with disease activity, this may be a result of trafficking from blood to target organs. Autopsy material reveals an influx of mononuclear T cells, particularly Th1 CD4 T cells and Tc1 CD8 T cells. Also seen are microthrombi and fibrin, possibly trapping viral antigens (39, 40). An excessive release of proinflammatory IL-12, interferon gamma (IFNγ), tumor necrosis factor (TNF) and IL-6 produce a “cytokines storm” which can lead to tissue damage and death (39, 40). Subsequent “control” of viral infection may be impaired due to immune exhaustion of the T cells (41), perhaps prolonging the clinical state in the face of other inflammation. In this later stage, treatment with methylprednisolone and/or anti-IL6 monoclonal antibodies may reverse the storm and slow down the damage (42).

An important factor determining T cell tropism is antigen concentration (43). Like other coronaviruses, there are several proteins involved in viral replication, the S or spike protein, the M or matrix protein, the E or envelope protein, and the N or nucleoprotein. Viral titer in the throat, peaks in the 1st week and declines by day 14. Antibody is documented in some by day 7 and most by day 14. They consist of IgM, IgA and IgG to spike or nucleoprotein antigens. The appearance of antibody did not preclude the ability to detect virus in some as they may not be neutralizing antibodies (44). Moreover, some individuals with mild SARS-CoV-2 infection developed T cell responses in the absence of antibody seroconversion (45). Experience derived from SARS and MERS showed that neutralizing antibody and B cell responses fade after 1–2 years (46). However, HLA-restricted central memory CD4 and effector memory CD8 T cell responses to spike proteins persist for several years (47, 48).

In children, adaptive responses are more Th2 polarized (49). This holds true for responses to respiratory viruses. When there is pulmonary pathology seen, airway CD8 Tc1 response is seen (50). Th2 responses downmodulate Th1 responses which likely induces tissue damage of infected cells. This Th2 predominant response would ameliorate the cytokine “storm” seen in adults and resulting in significant clinical disease. After infection with the SARS virus in children, high levels of IL-1 were seen in the 1st week. The traditional Th1 cytokines, IL-6 and TNF, that were high in adults with COVID-19, were not overly expressed at first and declined thereafter in children (51).

## Coinfections with Respiratory Viruses

Children commonly experience multiple viral infections, such as rhinovirus, RSV, and influenza, causing respiratory symptoms, including rhinorrhea in most, and often cough and gastrointestinal complaints (52). Previous infection or coinfection with these may modify the immune response to the COVID-19 virus. Children are frequently infected with multiple agents at the same time (53–55). However, there is a paucity of data related to coinfections due to shortage of viral transport media paired with lack of availability to make home-brew media based on CDC recommendations^1^, and so the true co-infection rates at this time require further study.

Seasonal human coronaviruses HCoV-229E, -NL63, -OC43, and -HKU1 contribute to a considerable share of upper and lower respiratory tract infection in children. The majority of children are seropositive for HCoV-229E and -NL63 by the age of 3.5 years (56, 57). Interestingly, there may be cross-reactive immunity between seasonal human coronaviruses and COVID-19. In one study of samples stored prior to the COVID-19 pandemic, nearly 50% of healthy adults were found to have SARS-CoV2-specific CD4 T cells (58). In this study, there were also SARS-CoV2-specific CD8 T cells in a smaller subset of COVID-19 unexposed adults. It is conceivable that these pre-existing SARS-CoV2-specific T cells contribute to an overly robust T cell response after COVID-19 infection leading to cytokine storm and severe disease. Young children may be naïve to seasonal human coronavirus strains or mount weaker T cell responses that cross-react with SARS-CoV2, ironically protecting them from cytokine storm and the inflammatory process associated with severe disease. Indeed, among children hospitalized in intensive care units, over half were above the age of 10 years (59), which parallels when we would expect higher seropositivity to seasonal human coronaviruses and immunologic maturity that has shifted from Th2 to Th1 predominance and stronger T cell responses.

Conversely, smaller studies have shown that children who develop multi-system inflammatory syndrome in children (MIS-C) were less likely to have antibodies to seasonal coronaviruses: this could be itself because MIS-C children represent an older age group, or because having these antibodies plays a role in controlling later hyperinflammatory COVID-19 associated complications (60). Interestingly, a study from Seattle Children's in the early pandemic showed a surprisingly low seroprevalence of SARS CoV2 antibody in children, although neutralizing antibody activity was detected in children in whom SARS CoV2 infection had not been suspected (61). Perhaps this is not surprising though given that we know in adults, detectable antibody is more likely to be seen those who are symptomatic (62). Moreover, some individuals with mild SARS-CoV-2 infection develop T cell responses in the absence of antibody seroconversion (45). In addition, a recent study examining immune responses in pediatric compared with adult patients with COVID-19 infection (and for pediatrics, acute COVID-19 and MIS-C patients were included) found that adults with COVID-19 were more likely to have neutralizing antibody titers compared with children, as well as lower IL-17A and interferon gamma responses, and children in this study did not have differences in anti-spike antibody titers when compared to adults (63). Of note, some of these studies so far have relatively small numbers and larger studies to clarify the immune response profile distinctions amongst children with acute COVID-19 compared with MIS-C compared with adults with acute COVID-19 (severe and less severe/asymptomatic) are needed, especially in the context of the COVID-19 vaccine development efforts.

## Spectrum of COVID-19 Manifestation in Children

In adults, coexisting health issues associated with inflammatory states such as obesity, type 2 diabetes, cardiac disease with impaired function, pulmonary insufficiency associated with exposure to respiratory toxins, liver disease facilitated by alcohol exposure, and declining renal function were associated with worse outcomes (64). Similarly, a recent report from pediatric ICUs in the United States revealed that among 48 COVID-19 infected children the mortality was 4% (59). Almost 90% of these children had preexisting comorbidities (59). Management of acute COVID-19 based on available evidence is reviewed in the Infectious Disease Society of America COVID-19 Guidelines (65).

However, the spectrum of disease in children continues to be described, and co-morbidities in children, as they are in adults, may not account for the entire picture. Basic knowledge of immunologic ontogeny, paired with what we have seen thus far with COVID-19, suggests that infected children develop varied manifestations of the COVID-19 infection: (1) asymptomatic infection, if innate immunity resolves upper respiratory infection; or (2) mild fever with upper respiratory symptoms; or (3) lower respiratory infection with some symptomatology which resolves after a few days. These would be expected with a Th2 preponderance, based on immunologic maturity. If the virus were allowed to disseminate to other tissue and the child developed adaptive responses to the virus over the following one or more weeks, the response to SARS-CoV-2 antigens might provoke a delayed inflammatory state. Therefore; (4) IgA immune complexes may result in systemic inflammatory responses that mimic Th1 responses and that also may produce a diverse set of syndromes that they are genetically at risk for.

## Later Manifestations of COVID-19

There have been recent individual reports of hyperinflammatory disease with parallels to other multisystem inflammatory disease syndromes in children, experienced by relatively small numbers of children with evidence of COVID-19 infection by PCR or antibody testing (66). The first and largest case series were published from the Italian, British, and American (New York) experiences, prompting emergency announcements regarding what is being termed as “Multisystem Inflammatory Syndrome in Children” or MIS-C, and case definitions released by the U.S. and European Centers for Disease Control as well as the WHO on May 14 and 15, 2020 (67–69). Many of these cases cluster at least a month after the peak of community transmission, with the vast majority testing SARS-CoV-2 IgG positive. The definitions comprise the clinical symptoms and lab findings of inflammation as well as evidence of, or exposure to, COVID-19. Abnormal labs include overall increased WBC, low lymphocytes, increased CRP, lower albumin, increased ferritin, increased troponin, elevated d-dimers, increased LDH, and abnormal troponin and BNP (67).

Children are presenting with a spectrum of manifestations with prolonged fevers in the absence of alternative diagnosis, and in worst cases, frank cardiogenic shock with myocardial dysfunction (70). There is no known predictive factor as to which children will have worse disease, and there may be host genetics at play as these cases were described in Europe and have not yet been described in East Asian countries (for example, China) that have gone through COVID-19 epidemic peaks (71). In addition, a recent small study from Paris reported MIS-C was more commonly seen in children of African ancestry (72). The initial reports of this condition from the U.K. showed this diagnosis also was frequent in children of Afro-Caribbean descent (68), and data from the Centers for Disease Control and Prevention suggest this condition is more commonly described in racial and ethnic minority (Hispanic and black) children (73). Moreover, we know MIS-C is a relatively rare complication, suggesting potential host genetic factors at play. In addition, we do know that acute COVID-19 is more commonly diagnosed in racial and ethnic minority children, and the more frequent occurrence of MIS-C in racial and ethnic minority populations may reflect factors that contribute to acute COVID-19 being also more frequent in these populations.

While there is data for treatment guidelines continues to be gathered at this time, the current consensus or recurring themes of treatment include treatment with immunomodulatory treatment (e.g., IVIG, steroids), anticoagulant therapy, frequent echocardiograms, and extreme caution with fluid resuscitation and IVIG administration if it is used. Indeed the American Academy of Pediatrics has published interim guidance at this time, and the American College of Rheumatology has the first set of published guidelines for this (74). Optimum follow-up for cardiac complications also requires further study (75). Other more specific immunomodulators have also been employed, including anti-IL-6 and anti-IL-1 inhibitors, with a preference toward the latter due to its safety profile and short half-life. Parallels between MIS-C and the hyperinflammatory response in the acute phase of COVID-19 in adults have been drawn, but whether the mechanisms are similar or different remains to be defined. Implications for vaccine development are also a concern given the severe post-inflammatory syndrome seen with these children.

## MIS-C, Other Inflammatory Childhood Diseases, and Parallels Drawn

The clinical phenomena of MIS-C shares features of other genetic, inflammatory and post-infectious childhood diseases, and in fact, calls to streamline the case definition have been made to avoid diagnostic overlap (76). Some of these syndromes share features of Monogenic Autoinflammatory Disease which is marked by overexpression of IL-1 and in some who develop Macrophage Activating Syndrome, overexpression of IL-18. A review of clinical and laboratory features referenced below and peculiar to each disease is summarized in Table 1.

**Table 1:** Characteristics of MIS-C and other Hyperinflammatory Syndromes.

	**MIS-C**	**KD**	**HLH**	**MAS**	**HSP**
**System/organ manifestations**					
Skin/rash	+	+	+		+
Kidney	+				+
GI tract	+				+
Vasculitis	?	Medium vessel			Small vessel
Central nervous system	+	+			
**Clinical labs**					
WBC	↓	↑			
Polymorphonuclear cells	↑	↑↑ (early)	↓	↓	
Lymphocytes	↓		↓	↓	
Platelets	↓	↑*↑↑*		↓	
RBC	↓ or normal		↓		
Albumin	↓		↓		
CRP	↑	↑	↑	↑	↑
D-Dimer	↑↑		↑↑	↑↑	
Ferritin	↑		↑*↑↑*		
Fibrinogen	↑		↓	↓	
Triglycerides	?		↑	↑	
Troponin	↑	↑ or normal			
BNP	↑↑	↑			
**Immune labs**					
IL-6	↑	↑	↑	↑↑	↑
IL-1	?	↑	↑	↑	
IL-8	?	↑			↑
IFNγ	↑	↑	↑	↑	
IgA	?	↑	↑		↑

### Kawasaki Disease

Kawaski Disease (KD) demonstrates some pathophysiologic features of COVID-19 related inflammation. KD-experts are very cautious to distinguish MIS-C from KD, though, and there remains the question of what exactly MIS-C is, as it is not KD itself. MIS-C occurs in older children (although there are possibly cases in adults) (77), and presenting symptoms include more prominent gastrointestinal symptoms and cardiac features, with parallel distinct laboratory features such as elevated ferritin, D-dimers, and triglycerides (67), which bear more resemblance to Macrophage Activation Syndrome (MAS) (71).

Clinical diagnostic criteria of KD include persistent fever, rash, cervical lymphadenopathy, bilateral conjunctival erythema, mucositis and peripheral swelling, which overlap with MIS-C. In KD, there are increased levels of IL-1, IL-6 and IL-8. Neutrophils predominate in the 1st week and may invade the wall of medium-sized arteries such as the coronaries. With time they are joined by macrophages, dendritic cells and CD8 T cells. Immune complexes are prevalent but there is little evidence for a major role in pathogenesis, and no target antigen has been identified reliably. Many different stimuli (e.g., commensal bacteria, mycoplasma, candida, different viral classes) have been suggested. Early treatment with high dose IVIG and/or steroid plus aspirin resolves fever and prevents coronary artery aneurysms.

Children who do not meet diagnostic criteria may have “Atypical” KD and are treated as if they are “classical,” with good resolution of the symptoms. The pathogenesis may share common mechanisms but also elements of macrophage activation, resulting in inflammatory cytokine release and thrombotic phenomena (78). However, atypical KD often affects older children. Their platelet counts, which continue to rise to over 500,000 in KD, are often low or normal in the atypical disease. In comparison, the ability of COVID-19 to produce a hypercoagulable state may allow for clots to develop without elevated platelet counts.

COVID-19 infection induces antibody production 2–3 weeks after infection, although these antibody levels are higher in symptomatic patients compared with asymptomatic, and fall within months of infection (62). Although most antigens typically produce IgM followed by IgG, this infection produces IgA at the time IgM is seen, and the response is stronger and more persistent than IgM (79). Patients with COVID-19 produce increased IgA-secreting cells following TLR9 stimulation (80). IgA complexes can promote proinflammatory cytokines (TNFa, IL-1b, IL-6, IL-23) through FCalphaR1-TLR (TLR 3,4,5) crosstalk via gene transcription of macrophages, monocytes and Kupffer cells (81). Such a process would result in Th1-like inflammation that occurs several weeks after COVID-19 infection. A mouse model of KD vasculitis demonstrated that intestinal permeability and IgA provoked an immune vasculitis linked to cardiovascular inflammation (82).

### Hemophagocytic Lymphohistiocytosis (HLH)

HLH, in its familiar form, is typically seen in children under 18 months and is associated with genetic abnormalities. It can occur sporadically at any age. Two-thirds of patients have genetic, autoimmune or cancer predispositions (83). Prolonged PTT with hypofibrinogenemia and increased fibrin-split products are associated with easy bruising. Evidence of hemophagocytosis in the bone marrow is pathognomonic. There are increased levels of soluble CD25 (IL-2) and CD163 (macrophage marker). This disorder may be a result of an infectious trigger such as COVID-19 re-exposure.

Some children have developed a late-onset purpuric skin lesion in the lower extremities. While this may represent easy bruising, it also resembles the vasculitis rash of Henoch-Schonlein Purpura (HSP) in which there are deposits of IgA and IgG complexes in the affected area. There are also mesenteric areas with the same complexes, causing pain and perhaps diarrhea. The COVID-19 individuals also have enteric symptomatology and renal issues such as hematuria and proteinuria. There are likely genetic factors that play a role in HSP, particularly MHC class 2 and polymorphisms in the renin-angiotensin system, which may parallel COVID-19 pathophysiology (84). In contrast to some other inflammatory conditions, HSP doesn't respond well to corticosteroids but does improve with colchicine, a macrophage stabilizer.

Some children with MIS-C have developed macrophage-activated phenotypes, seen in HLH and MAS. In a multicenter study of 362 patients which predates COVID-19 and which aimed to describe MAS, symptoms included: fever (95%), hepatomegaly (70%), splenomegaly (58%), cardiac involvement (26%), hemorrhagic manifestations (20%), renal involvement (15%) and CNS symptomatology. Increased levels of ferritin, D-dimer, ALT, triglycerides and LDH are seen (85). Platelet counts and albumin levels are depressed. Treatment consists of anti-inflammatory drugs such as corticosteroids, cyclosporin, anti-IL1 agents and etoposide. Plasma exchange is often needed. In a quarter of patients, infection with EBV or other Herpesviruses may be a trigger (85).

### Post-streptococcal Acute Rheumatic Fever (ARF), an Historic Reference

ARF set a precedent for the development of a systemic disease that occurred weeks after an initial infection with a pathogen, and comparisons to COVID-19 and its associated syndromes have been explored (86). ARF followed a pharyngeal infection with a common bacterial pathogen, the Group A Streptococcus. The initial infection was frequently asymptomatic. In a few individuals (~1/1,000) over age 2, fever developed about 2 weeks later. In one study, the majority presented with pain in the joints, manifested as arthritis in 81% and arthralgia in 15%. Four percent presented with congestive heart failure. Carditis developed in 42%, with a high incidence of pericarditis (6%) and congestive heart failure (15%) and a mortality of 2% (87). In another study, although antibodies to streptococci developed, and may have recognized myosin, the disease was due to T cell stimulation (88). The M protein of certain streptococci can produce superantigen-like stimulation of T cells, resulting in cytokine release (89–91). Treatment of ARF with anti-inflammatory doses of aspirin or corticosteroids resolved the inflammation. If treatment was initiated early enough, the arthritis and/or carditis resolved. If treatment occurred later, valvular damage was irrevocable. There were instances of recurrent inflammation after exposure to group A streptococcus, which was prevented by antibiotic prophylaxis. When recurrence did occur, there was a reprise of the original syndrome of carditis or arthritis. This suggests a likely genetic predisposition, which is supported by twin studies showing a high concordance of ARF in monozygotic twins (92). Genetic and genome-wide association studies have reproducibly found a link between ARF and the HLA locus on chromosome 6 as well as the immunoglobulin heavy chain locus which includes *IGHV4-61* (93).

ARF, while not uncommon prior to 1970 in the USA, has become uncommon since then. Its rarity in children under 2 years was thought to be related to their relative inability to develop sufficiently high levels of Th1 responses. The delay from primary SARS-CoV-2 infection to disease is reminiscent of ARF in the past, which also occurred in a small fraction of at-risk individuals.

#### Other Potential Host/Genetic Factors

The first genetic association studies looking at host genetic factors in COVID-19 patients published as preprints have suggested a link between severe disease and certain ABO blood types including A and B (32) and Rh positivity (94). Furthermore, preliminary results of a genome-wide association study of Italian and Spanish patients found not only an association between the ABO locus (chromosome 9q34) and more severe disease, but also a gene cluster on chromosome 3p21 which includes a gene that encodes a proline transporter that interacts with ACE2 (*SLC6A20*) as well as two chemokine genes, CC-motif chemokine receptor 9 (*CCR9*) and the C-X-C motif chemokine receptor 6 (*CXCR6*) (95). It has yet to be determined if either of these loci will play a role in the development of MIS-C or severe disease in children. There are several ongoing international collaborative efforts (including the COVID Human Genetic Effort and the COVID-19 Host Genetics Initiative) which aim to uncover common and rare host genetic variation that contributes to either COVID-19 infection severity or the development of the rarer complications and clinical syndromes, including those that seem to affect only younger patients and children (96, 97). Indeed, there is now at least one convincing report describing 2 pairs of brothers from the Netherlands who all developed severe COVID-related disease and after whole exome sequencing were found to have loss-of-function variants in the X-linked gene *TLR7* which encodes toll- like receptor 7, an inducer of the interferon I and II responses in the innate immune system (98).

As we try to establish the host genetic factors underlying the emerging pediatric COVID-19-related syndromes like MIS-C, it may be helpful to review what is known about the genetic etiologies behind HLH and KD (Table 2). Indeed, COVID19-related illnesses in children (in particular MIS-C) may share patho-etiological origins, or genetic susceptibilities, with other pediatric hyperinflammatory syndromes. HLH is usually categorized into a primary (or familial) form and a secondary (or acquired) form (99). The former is a classic Mendelian disorder, inherited in an autosomal recessive fashion^2^. There is, however, also evidence that heterozygotes (carriers) of pathogenic alleles in one of the known HLH genes might also develop HLH (100). The pathological basis of HLH (or the macrophage-activating syndrome as it is sometimes called when it occurs in a patient with an antecedent rheumatological disease) is the disordered loading, moving, priming and/or docking of the cytotoxic T-lymphocyte's toxic granules leading to target cell death and overproduction of cytokines, in particular IFNγ and TNFα (101). The products of the genes that are associated with HLH all seem to play a role in this process and therefore may be potential susceptibility loci in those with severe COVID-19-related syndromes, especially wherein there is immunological and clinical evidence of an HLH-like process.

**Table 2:** Primary Hemophagocytic Lymphohistiocytosis and Kawasaki Disease-Associated Genes.

**Phenotype**	**Gene name**	**Gene product's putatuve function, mendelian disorder association (NCBI Gene, OMIM)**
**Primary hemophagocytic lymphohistiocytosis**		
Familial HLH	*PRF1*	Forms membrane pores that allow the release of granzymes and subsequent cytolysis of target cells. Hemophagocytic lymphohistiocytosis, familial, 2.
	*STX11*	Implicated in the targeting and fusion of intracellular transport vesicles. Hemophagocytic lymphohistiocytosis, familial, 4.
	*STXBP2*	Involved in intracellular trafficking, control of SNARE (soluble NSF attachment protein receptor) complex assembly, and the release of cytotoxic granules by natural killer cells. Hemophagocytic lymphohistiocytosis, familial, 5.
	*UNC13D*	Appears to play a role in vesicle maturation during exocytosis and is involved in regulation of cytolytic granules secretion. Hemophagocytic lymphohistiocytosis, familial, 3.
Primary Immunodeficiencies associated with HLH	*AP3B1*	May play a role in organelle biogenesis associated with melanosomes, platelet dense granules, and lysosomes. Hermansky-Pudlak syndrome 2.
	*BIRC4*	Functions through binding to TNF receptor-associated factors TRAF1 and TRAF2 and inhibits apoptosis induced by menadione, a potent inducer of free radicals, and interleukin 1-beta converting enzyme. Also inhibits at least two members of the caspase family of cell-death proteases, caspase-3 and caspase-7. X-linked lymphoproliferative syndrome.
	*CD27*	Required for generation and maintenance of T cell immunity. Binds ligand CD70, plays key role in regulating B-cell activation and immunoglobulin synthesis. Transduces signals that lead to the activation of NF-kappaB and MAPK8/JNK. Lymphoproliferative syndrome 2.
	*ITK*	Encodes an intracellular tyrosine kinase expressed in T-cells. Lymphoproliferative syndrome 1.
	*LYST*	regulates intracellular protein trafficking in endosomes, and may be involved in pigmentation. Chediak-Higashi syndrome.
	*RAB27A*	May be involved in protein transport and small GTPase mediated signal transduction. Griscelli syndrome type 2.
	*SH2D1A*	Plays a major role in the bidirectional stimulation of T and B cells. Lymphoproliferative syndrome, X-linked 1, or Duncan disease.
**Kawasaki disease**		
Development of KD	*ABCC4*	Member of the superfamily of ATP-binding cassette (ABC) transporters, MRP subfamily involved in multi-drug resistance. Plays a role in cellular detoxification as a pump for its substrate, organic anions. May also function in prostaglandin-mediated cAMP signaling in ciliogenesis.
	*CD40*	Part of TNF-receptor superfamily. Receptor on antigen-presenting cells, essential for mediating variety of immune and inflammatory responses including T cell-dependent immunoglobulin class switching, memory B cell development, and germinal center formation. Hyper-IgM immunodeficiency, type 3.
	*FCGR2A*	Immunoglobulin Fc receptor gene, found on the surface of many immune response cells. Cell surface receptor found on phagocytic cells such as macrophages and neutrophils, involved in the process of phagocytosis and clearing of immune complexes.
	*TLR6*	Plays a role in pathogen recognition and activation of innate immunity. Recognizes pathogen-associated molecular patterns (PAMPs) that are expressed on infectious agents, and mediate the production of cytokines necessary for the development of effective immunity.
Resistance to IVIG +/- CAD development in KD	*ITPKC*	Encodes a member of the inositol 1,4,5-trisphosphate [Ins(1, 4, 5)P(3)] 3-kinase family of enzymes that catalyze the phosphorylation of inositol 1,4,5-trisphosphate to 1,3,4,5-tetrakisphosphate+/NFAT pathway
	*CASP3*	Cysteine-aspartic acid protease that plays a central role in the execution-phase of cell apoptosis
	*ORAI1*	Membrane calcium channel subunit that is activated by the calcium sensor STIM1 when calcium stores are depleted. Primary way for calcium influx into T-cells. Immune dysfunction with T-cell inactivation due to calcium entry defect, type 1.

The host genetic factors contributing to the development of KD are less clear. The currently accepted hypothesis is that KD is a multifactorial disorder that develops in a genetically susceptible child after an environmental/infectious trigger. So far, no one has established any Mendelian forms of KD; KD does not have a Mendelian entry in OMIM. Twin studies in KD are conflicting but suggest some concordance in the development of KD-related vasculitis (102). There are also reports of familial clustering of KD in Japan (103). While the genes implicated in monogenic forms of HLH tend to converge on a single T-cell process, the genes so far associated with KD and its complications are more biologically diverse and roughly fall into four categories: T-cell activation, B-cell signaling, cellular apoptosis and dysfunctional transforming growth factor beta signaling (104). Furthermore, there is at least one report of a specific viral trigger (EBV) leading to KD in a patient with Familial Mediterranean Fever due to mutations in *MEFV* which encodes pyrin, a protein involved in the innate immune response (105). Interestingly, a rare complication of KD is HLH itself (106), suggesting that, at least in some, the pathological mechanisms of both disorders may overlap.

## Conclusion

COVID-19 infection in children is markedly different from adults in a number of ways, including differences in transmission itself, as well as differences in severity and pathogenesis including viral genetic diversity, sex-related-impacted protease and viral susceptibility differences, and age-related and potentially genetic innate and adaptive immune response differences. Parallels to other hyperinflammatory syndromes and comparisons in the context of some of these factors may also continue to lead to understanding pediatric susceptibility to COVID-19 disease and its associated syndromes. Understanding transmission and pathogenesis in children is essential to optimizing the care of children, and also very practically, implementing strategies to allow children to attend school and adopt back to some degree of normalcy.

Children have relatively little symptomatology upon acute infection. This may be a consequence of developmental “immunodeficiency” resulting from a Th2 polarization that starts *in utero* and is maintained for most of the first decade of life. Th2 responses may suppress Th1 proinflammatory processes seen in adults and are responsible for some of the increasingly severe symptomatology in advancing age. Th2 responses also allow for improved antibody responses, although the contribution of antibody responses to pathology compared with control of infection remains to be clarified in adults and children. With maturation of this process in the second decade of life, increasing symptoms are sometimes seen with acute infection. In a very small subset of children, a diffuse set of inflammatory syndromes are seen several weeks after infection with COVID-19. These entities may develop in a genetically vulnerable population with susceptibilities that are similar to those already illuminated for other inflammatory syndromes (e.g. Kawasaki Disease, HLH/MAS and HSP). Another possibility is that these inflammatory syndromes occur as a consequence of IgA complexes resulting from the Th2 environment or as a result of a Th1-like response to COVID-19 antigens that were slow to develop after infection.

Fortunately, children respond to typical treatment for the syndromes. However, as children are certainly not just little adults, we need to learn and model through vaccines or immune-modulatory treatments how children handle this infection so much better than we do. We as adults can learn from our children–as we always should.

## Author Contributions

AK, CH, BK, and WB contributed wrote, reviewed, edited the manuscript, and tables. WB conceived the paper. All authors contributed to the article and approved the submitted version.

## Conflict of Interest

The authors declare that the research was conducted in the absence of any commercial or financial relationships that could be construed as a potential conflict of interest.

## Footnotes

^1^CDC. Available online at: https://www.cdc.gov/coronavirus/2019-ncov/downloads/Viral-Transport-Medium.pdf (accessed May 20, 2020).

^2^OMIM: Available online at: https://www.omim.org/entry/613101 (accessed May 16, 2020).
